# Increase in Levels of BDNF is Associated with Inflammation and Oxidative Stress during Cardiopulmonary Bypass

**Published:** 2008-09

**Authors:** Sébastien Amoureux, Pierre Sicard, Claudia Korandji, Angélique Borey, Salima Benkhadra, Anabelle Sequeira-Le Grand, Catherine Vergely, Claude Girard, Luc Rochette

**Affiliations:** 1 *LPPCE, IFR Santé STIC 100, Facultés de Médecine et de Pharmacie, Dijon, France;*; 2 *Département d’Anesthésie Réanimation, CHRU Le Bocage, Dijon, France;*; 3 *Centre de Cytométrie en Flux, IFR Santé STIC 100, Facultés de Médecine et de Pharmacie, Dijon, France*

**Keywords:** BDNF, cardiopulmonary bypass, oxidative stress, inflammation, cardiac surgery

## Abstract

Cardiopulmonary Bypass (CPB) is thought to generate reactive oxygen species associated with a systemic inflammation and neurotrophins seem to be involved in cardiovascular inflammatory reactions. The aim of this study was to determine the impact of CPB on plasma neurotrophins levels and to appreciate the links existing between inflammation, oxidative stress and neurotrophins. Blood samples were taken from 27 patients undergoing cardiac surgery: before CPB, during ischemia and at reperfusion under CPB. Oxidative stress was evaluated using an Electron Spin Resonance technique by superoxide detection, and antioxidant defences by measurement of Endogenous Peroxidase Activity (EPA). The evolution of two neurotrophins: Brain Derived Neurotrophic Factor (BDNF) and Nerve Growth Factor (NGF) was assessed with an ELISA method. An inflammatory index was determined by a multiplex flow cytometry method. The inflammatory index showed that MCP-1, P-selectin, t-PA and interleukins 6, 8 and 10 levels increased during CPB (*p*<0.05). Superoxide production and EPA were higher during ischemia and reperfusion than before CPB (*p*<0.05). BDNF plasma levels were higher at reperfusion (*p*<0.05). NGF levels did not change. Our study shows an increase of BDNF levels, associated with an inflammatory phenomenon and a redox modification, in the plasma of patients undergoing cardiac surgery under CPB. The role played by this neurotrophin in this complex situation still needs to be elucidated, in particular its cellular origin. It is also necessary to understand whether BDNF has a beneficial or deleterious effect during CPB.

## INTRODUCTION

Over the years, Cardiopulmonary Bypass (CPB) has been improved, but still shows many sides effects. Several organs can be affected (Multi Organ Dysfunctions (MOD)): kidney, lungs, heart and central nervous system. Many studies showed that the walls of the CPB circuit activate white cells, platelets and the complement system. Activated leukocytes release cytotoxic agents into the bloodstream, which is associated with the deleterious effects of oxidative stress during CPB (1, 2, 3). These processes may be involved in the development of cardiovascular injuries found in MOD and in particular in cardiovascular injuries (4).

In this context of inflammation that occurs in cardiovascular and neurovascular systems, different studies have suggested that neurotrophins are involved (5, 6). These molecules are neurological growth factors, but they are also involved in the development of vascular tissues, such as endothelium (7, 8). Recent studies (9, 10) have underlined the possible implication of neurotrophins and specifically Brain Derived Neurotrophic Factor (BDNF) in cardiovascular diseases and myocardial injury: two situations in which inflammation is involved. Cardiovascular disorders such as ischemia and necrosis are connected with high levels of oxidative stress, and BDNF itself shows capacities of activation of oxidative enzymes like NAD(P)H oxidases (11). Other works have focused on another neurotrophin: Nerve Growth Factor (NGF). NGF appeared to be involved in endothelial migration after inflammation (12), and this neurotrophin shows indirect antioxidant effects through the activation of superoxide dismutase and peroxidases (13). The role of neurotrophins in inflammation/oxidative stress remains to be elucidated.

The present study was designed to assess the evolution of plasma neurotrophin concentration in a highly inflammatory and oxidative situation such as CPB. Our hypothesis is that BDNF and NGF may be involved in the modulation of inflammation and oxidative stress during CPB. To confirm this, our objectives were 1) to investigate the inflammatory and redox profile of patients undergoing cardiac surgery under CPB and 2) to determine the behaviour of two neurotrophins, BDNF and NGF in patients’ blood during the procedure.

## METHODS

### Study population and design

Our study complies with the Declaration of Helsinki. After gaining approval of the local research ethics committee and obtaining the patients’ informed written consent, 27 patients undergoing coronary artery re-vascularisation or valve replacement (alone or combined) under CPB were included in this prospective study.

The following criteria led to the exclusion of patients from the protocol: surgical emergencies, a past medical history of heparin, current chronic inflammatory pathologies, coagulation troubles (except for heparin or aspirin treatment) and aneurismectomy-associated surgery. All routine cardiac medication was continued until the morning of surgery.

Anticoagulation therapy was stopped 10 days before surgery. Patients were pre-medicated with midazolam (0.1 mg/kg) orally plus hydroxyzine (1 mg/kg) 90 min before anaesthesia.

Before the induction of anaesthesia, complete hemodynamic monitoring was set up in the operating room. Monitoring consisted of a pulmonary artery catheter for the continuous measurement of cardiac output and SvO_2_ through the right internal jugular vein, a peripheral 14 Gauge venous catheter, a 20 Gauge arterial catheter, pulse oxymetry and a 5-lead electrocardiogram (ECG). The hemodynamic profile consisted in heart rate, mean pulmonary artery pressure, pulmonary capillary wedge pressure, cardiac index, and systemic vascular resistance using a Baxter Vigilance monitor.

Induction of anaesthesia was performed with intravenous midazolam (0.02 mg/kg), sufentanil (0.2 μg/kg) and hypnomidate (0.3 mg/kg). After verifying correct manual ventilation, cisatracurium dibesylate (0.15 mg/kg) was injected. Patients were orally intubated and ventilated with FiO_2_: 0.4. Anaesthesia was maintained with sufentanil and cisatracurium as required and inhaled isoflurane. After CPB weaning, isoflurane inhalation was stopped and intravenous propofol relay was established (1.5 mg/kg/h).

After intravenous injection of heparin (300 IU/kg), CPB was achieved in a standard fashion. A non-pulsatile pump was primed with Ringer lactate solution (1,000 mL) and hydroxy-ethyl starch (500 mL). The CPB flow rate was maintained between 2.4 L/min.m^-2^ (37°C) and 1.7 L/min.m^-2^ (28°C). Moderate body hypothermia (32°C) was used. An anterograde cardioplegia catheter was inserted into the ascending aorta and a retrograde cardioplegia canula was placed in the coronary sinus. After applying aortic cross-clamping (ACC), topical slush was applied to the heart and 500 mL of St Thomas’ Hospital cardioplegic solution was infused. Infusions were repeated every 30 min of clamping resulting in 1,500 to 2,000 mL of total cardioplegia. Re-warming began 10 min prior to aortic unclamping and 100 mL of mannitol solution (20%) was infused. Reperfusion on CPB was continued for 25-40 min after clamp removal. Finally, CPB was discontinued and protamine given for heparin reversal at the dose of 450 IU/kg. Patients benefited from blood infusions in case of severe anaemia (blood hematocrit <22% during CPB or <26% after CPB).

### Patients’ blood Samples

**Figure 1:**
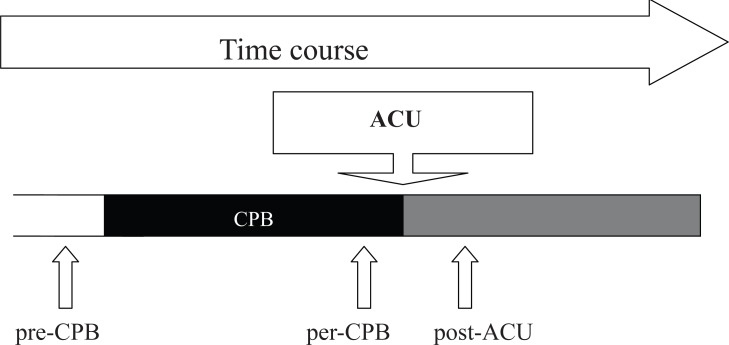
Chronology of plasma samples during procedure. 3 samples were taken; the first one pre-CPB was taken after anaesthesia induction before beginning of Cardiopulmonary Bypass (CPB), and represented the reference sample; the second sample per-CPB was taken 15 minutes before Aortic Cross Unclamping (ACU), and represented ischemia sample; and the third sample post-ACU was taken 15 minutes after ACU, and represented the reperfusion sample.

Three 10 millilitres arterial blood samples were taken from the patients following sample chronology shown in Figure 1: the first one (pre-CPB) was taken after induction of anaesthesia; the second one (per-CPB) was taken ten minutes before aortic cross unclamping (ACU); and the last one (post-ACU) was taken under CPB ten minutes after ACU. Blood was drawn into heparin tubes and immediately centrifuged at 3000 rpm for 10 minutes. One hundred microliters of plasma were taken for immediate use for Electronic Spin Resonance (ESR) assays; the rest of the plasma was stored at -80°C.

### Measurement of Inflammation Markers

To determine the inflammation profile of patients, multiplex flow cytometry was performed. A “Human Cardiovascular 7plex” (Bender Medsytems, Vienna, Austria) was used to measure plasma concentrations of 7 markers: CD40 Ligand (CD40 L), P-selectin, tissue Plasminogen Activator (tPA), Vascular Cells Adhesion Molecule 1 (VCAM-1), interleukin 6 (IL 6), interleukin 8 (IL 8), and Monocyte Chemoattractant Protein 1 (MCP-1). Interleukin 10 was also measured using a simplex Flowcytomix kit® (Bender Medsytems, Vienna, Austria). All assays were performed following manufacturers’ instructions, and were read using a flow cytometer BD LSRII®.

### Electronic Spin Resonance Spectroscopy

1-hydroxy-3-carboxy-pyrrolidine (CP-H; 100 μM; Noxygen, Germany) is a spin probe that can be oxidized by superoxide and peroxynitrite and becomes the radical CP^.^. This radical presents paramagnetic properties. To avoid metal-induced oxidation of CP-H, 1 mM of deferoxamin (Sigma, France) was added to the solution (14). Immediately after collection of a sample plasma, CP-H was added. The probe is oxidized by superoxide produced in the medium during incubation for 2 hours at 37°C. Twenty microliters of each sample was transferred into a quartz capillary (1mm i.d.) for analysis. All ESR spectra were recorded at room temperature on an ESP 300 E X-band spectrometer (Bruker, Wissembourg, France), using a TMH cavity. The height of the characteristic triplet of CP^•^ was measured and expressed in arbitrary units (AU). All results are given as percentages of the signals measured for the pre-CPB samples.

### Measurement of Endogenous Peroxidase Activity (EPA)

High amounts of peroxidases indicate either production of prooxidative substances by the patient or impaired consumption of antioxidants. To assess antioxidant defences, EPA was measured on patients’ plasma using a kit (Labor Diagnostika Nord, Nordhorn, Germany). It is a colorimetric test for quantitative determination of endogenous peroxidase activities in freshly prepared serum or EDTA-plasma samples. Assays were performed in polystyrene 96-well plates and were read at 450 nm. The concentrations of standards are given in peroxidase units. The results are expressed as mIU/mL and are corrected by hematocrit.

### Measurement of Neurotrophins

BDNF and NGF were measured on sampled plasma using ELISA assays (Promega, USA) following the manufacturer’s instructions. Assays were performed on polystyrene 96-well plates. The plates were read at 450 nm. The results are the means of duplicated measurements, and expressed as pg/mL and are corrected by hematocrit.

### Data analysis

One way ANOVA tests were used for statistical analysis of differences between pre-bypass values and time points. Data not normally distributed were analysed using Dunn’s multiple comparisons tests. Significance levels for acceptance were *p*<0.05. The results in Tables and text are presented as means ± standard error (SEM).

## RESULTS

### Patients

The 27 patients included in our work were 79 ± 4 years old. There were 16 males (59%). Thirteen patients underwent valve replacement (alone or combined), 10 underwent coronary artery re-vascularisation, and 4 patients underwent the two procedures combined. Sixteen patients suffered from hypertension, 9 from dyslipidemia, 3 from type I diabetes and 4 from type II diabetes. Patients showed a left ventricular ejection fraction of 60.5 ± 3.0%. CPB lasted 101 ± 4 minutes for a mean ACC time of 76 ± 4 minutes. Following the surgery, the mean period in the Intensive Care Unit (ICU) was 2.9 ± 0.3 days. The results are shown in Table 1.

**Table 1:** Population Characteristics

Population	
Patients	27
Males	16 (59%)
Females	11 (41%)
Age (years)	79.4 ± 4.1
Disease History and Cardiac Function	
Hypertension	16 (59 %)
Dyslipidemia	9 (33 %)
Type I Diabete	3 (11 %)
Type II Diabete	4 (15 %)
LVEF	60.5 ± 3.4 %
Surgery procedure and Anaesthesia	
Coronary Artery Re-vascularisation	10 (37 %)
Valvular Replacement	13 (48 %)
Coronary Artery Re-vascularisation and Valvular Replacement	4 (15 %)
ACC Time (min)	76.7 ± 4.4
CPB Time (min)	101.4 ± 4.2
Post-operative ICU time (days)	2.85 ± 0.3

ACC, Aorta Cross Clamping; CPB, Cardiopulmonary bypass; ICU, Intensive Care Unit.

### Inflammation Markers

Flow cytometry assays showed that CPB increased plasma concentrations of some of the studied inflammation markers: MCP-1; P-selectin; t-PA, IL 6; IL 8 and IL 10 (*p*<0.05; comparisons: per-CPB *vs.* pre-CPB and post-ACU *vs.* pre-CPB). VCAM-1 and CD40L levels remained unchanged during surgery. The results are shown Table 2.

**Table 2:** Inflammatory markers time course in plasma of patient undergoing surgery under Cardiopulmonary Bypass (CPB)

	Pre-CPB	Per-CPB	Post-ACU

MCP-1 (pg/mL)	355.92 ± 55.88	1196.23 ± 218.25^a^	1238.52 ± 165.67^b^
P-selectin (ng/mL)	103.05 ± 12.97	121.37 ± 13.08^a^	1140.09 ± 12.98^b^
t-PA (pg/mL)	1331.45 ± 168.78	3788.32 ± 281.85^a^	4037..83 ± 490.52^b^
IL 6 (pg/mL)	18.09 ± 7.44	75.24 ± 17.88^a^	119.61 ± 25.26^b^
IL 8 (pg/mL)	19.80 ±7.46	254.26 ± 126.23^a^	305.85 ± 166.15^b^
IL 10 (pg/mL)	43.52 ± 24.07	475.95 ± 215.67^a^	319.63 ± 97.78^b^
VCAM 1 (pg/mL)	474.34 ± 182.36	541.30 ± 58.59	500.33 ± 30.79
CD40 L (ng/mL)	914.18 ± 100.39	1084.63 ± 93.68	1062.33 ± 112.89

ACU, Aortic Cross Unclamping; IL, interleukin; MCP-1, Monocyte Chemoattractant Protein 1; t-PA, tissular Plasminogen Activator; VCAM-1, Vascular Cells Adhesion Molecule 1; CD40 L, CD40 Ligand.

a per-CPB vs pre-CPB, *p*<0.05;

b post-ACU vs pre-CPB, *p*<0.05.

### Oxidative profile

**Figure 2:**
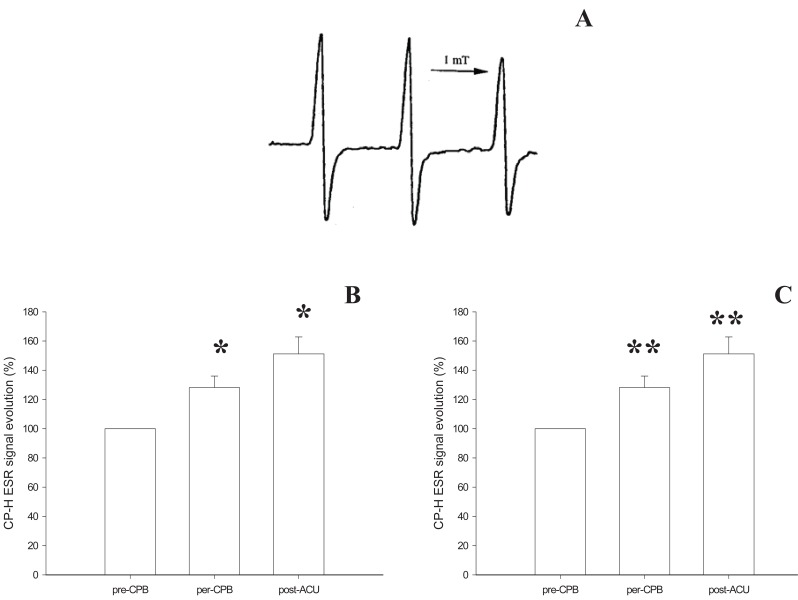
Oxidative profile evolution of patients undergoing cardiac surgery under Cardiopulmonary Bypass (CPB). **A**, represents the CP^•^ (CPH (1-hydroxy-3-carboxy-pyrrolidine) oxidation product) characteristic ESR triplet signal recorded at room temperature on an ESP 300 EX-band spectrometer; **B**, represents this signal evolution in plasma of the patients. Patients presented an increase in plasma capacities to produce superoxide that oxidize CPH during CPB that continued after Aortic Cross Unclamping (ACU) (*p<0.05; Comparison: per-CPB *vs.* pre-CPB and post-ACU *vs.* pre-CPB). Results are expressed as a percentage of pre-CPB ESR signal measured in plasma after 1h incubation with CP-H (1 mM); **C**, shows the increase in Endogenous Peroxidase Activity (EPA; expressed in mIU/mL) during CPB (**p<0.01; Comparison: per-CPB *vs.* pre-CPB and post-ACU *vs.* pre-CPB).

The capacity of the plasma to oxidize CP-H was increased with CPB: the ESR signal was 28.1 ± 7.9% (*p*<0.05) higher during heart ischemia than before CPB rising to 51.1 ± 11.7% (*p*<0.05) higher for the reperfusion period (Figure 2B).

This increase was associated with an increase in EPA: 12.14 ± 3.86 mIU/mL at anaesthesia induction rising to 33.23 ± 4.20 mIU/mL 15 minutes before ACU and 37.16 ± 1.64 mIU/mL 15 minutes after (*p*<0.01) (Figure 2C). This increase indicated stimulation in some of the antioxidant enzymatic defences.

### Neurotrophins measurement

ELISA assay in patients’ plasma showed that BDNF concentrations increased during surgery under CPB. Start levels of BDNF at the first sample time (pre-CPB), were 130.98 ± 24.39 ng/mL increasing to 242.16 ± 64.12 ng/mL at the second time (per-CPB) before falling back to 216.15 ± 24.39 ng/mL at the last one (post-ACU). This increase in BDNF is statistically significant (*p*<0.05).

**Figure 3:**
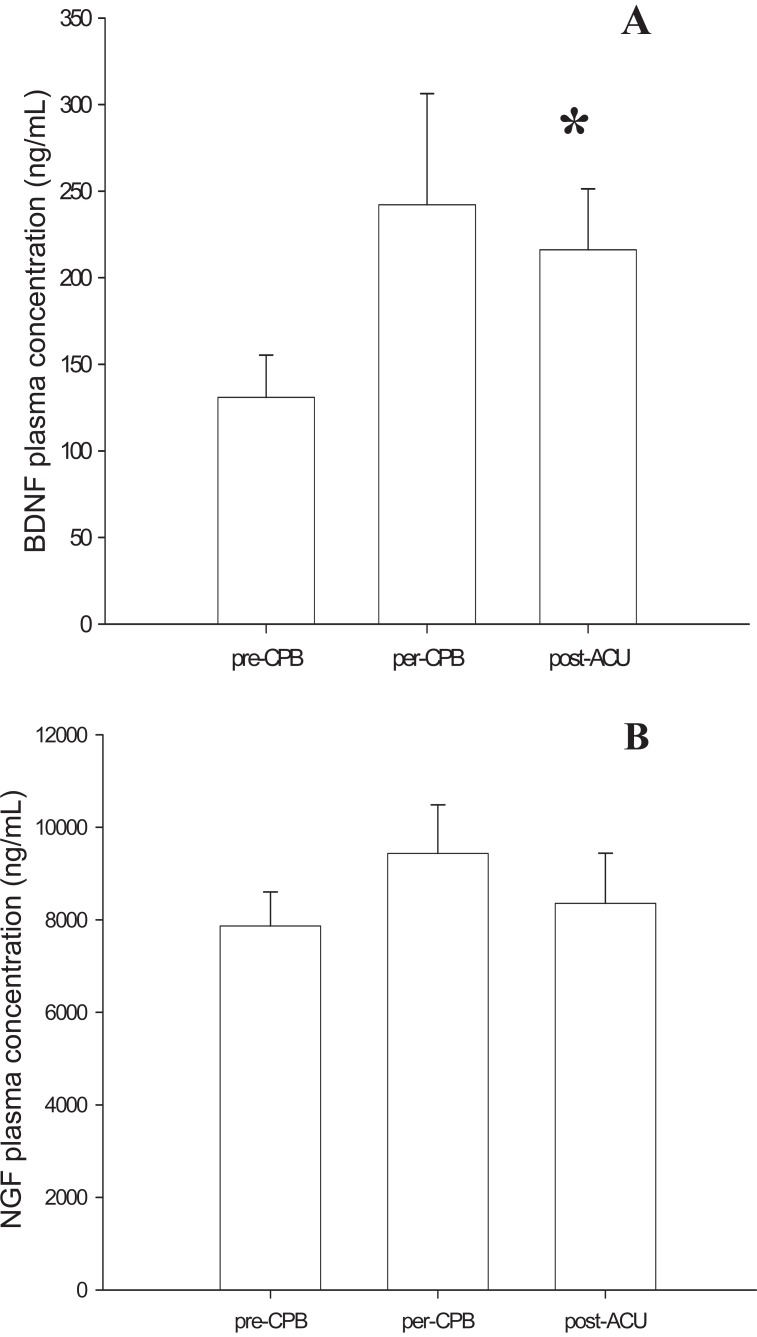
Neurotrophin concentration in plasma of patients undergoing surgery under Cardiopulmonary Bypass (CPB). A, During procedure, Brain Derived Neurotrophic Factor (BDNF) levels (expressed in ng/mL) increased in plasma before and after Aortic Cross Unclamping (ACU) (*p< 0.05; Comparison: post-ACU *vs.* pre-CPB); B, In the same time, Nerve Growth Factor (NGF) levels (expressed in ng/mL) didn’t show any variations during surgery under CPB.

NGF concentrations did not change during the procedure. They were 7866 ± 736 ng/mL before CPB, 9435 ± 1048 ng/mL during ischemia (per-CPB) and 8357 ± 1083 ng/mL at reperfusion (post-ACU) (Figure 3).

## DISCUSSION

The main finding in our study is the rise in plasma concentrations of BDNF during cardiac surgery under CPB. We observe that this evolution of BDNF plasma concentration is associated with an inflammatory phenomenon in patients’ blood during this procedure. This neurotrophin is involved in many pathological phenomena in the cardiovascular system (5, 10). Indeed different studies showed that an increased level of BDNF appears to be linked to cardiac injuries. Ejeri *et al*. (9) found the neurotrophin in atherosclerosis lesions: BDNF was present in vascular smooth cells, infiltrated macrophages and platelets. In another model, Cai *et al*. (10) suggested that age-associated changes in BDNF- receptor Trk B pathways may predispose the aging heart to increased injury after acute myocardial infarction, potentially contributing to the increased severity of cardiovascular disease in older patients.

In these different cases, BDNF is connected to inflammatory mechanisms and in particular during an ischemia/reperfusion sequence (15). Linker *et al*. (6) pointed out that BDNF can be synthesized in lymphocytes, polynuclear cells, macrophages and microglia. This neurotrophin and its receptor Trk-B are involved in inflammatory mechanisms and in immune regulation. Recently, Trk-B and BDNF were detected in the cardiovascular system: they may be involved in perivascular impairment linked to sympathetic dysfunction associated with inflammation (16).

NGF, another neurotrophin, did not show any variation in our patients’ plasma during surgery under CPB. Yet, in several works, it has been reported that this growth factor could be involved in the early phase of cardiac surgery accompanying the inflammatory phenomena occurring in the cardiovascular system. An *in vitro* study showed the capacity of NGF to induce proliferation of endothelial cells (12). These cells, after inflammatory stimulation via interleukin 1 β, secreted NGF.

This present study confirms the previous work of our group (3, 4) showing that cardiac surgery under CPB is the origin of a severe systemic inflammatory syndrome, and provides more precise data. Many works have shown that the non-biological wall of the CPB circuit activates blood cells: leukocytes and platelets (2, 17, 18). These cells release pro-inflammatory molecules: both cellular and non-cellular responses participate in the post-operative side-effects that occur after open heart surgery leading to MOD. In our work, IL 6 and IL 8, released by immune cells, are both increased during aortic cross clamping and this rise continues throughout the reperfusion period. The augmentation of MCP-1, t-PA, and P-selectin on endothelial cell surfaces confirms macrophage activation and platelet aggregation in the bloodstream. Several works have already highlighted the role these cytokines play in myocardial injuries due to activated immune cells (19, 20).

The blood cell activation and inflammation observed during surgery under CPB are thought to produce reactive oxygen species (ROS). Our work found modifications in patients’ plasma redox capacities under CPB. Enhancement of the CP^•^ signal measured during the procedure is a witness of the plasma’s capacity to generate ROS and particularly superoxide and peroxynitrite, which oxidize CP-H (21). This spin probe can be oxidized by different ROS that are produced *in vivo* by endogenous enzymes such as NAD(P)H oxidases (22). The oxidative capacity in plasma is enhanced during the ischemia period (per-CPB sample) and is increased further at reperfusion (post-ACU sample). The implication of ROS in side-effect mechanisms associated with CPB has been suspected for a long time and the production and detection of ROS during reperfusion have been evaluated in different circumstances (23, 24, 25). Our present results confirm previous work by Clermont *et al.* (3), in which the α-phenyl-*N-tert-*butylnitrone (PBN) ESR signal was increased by surgery and ischemia/reperfusion. PBN is a spin trap that reflects the presence of ROS in the bloodstream during the procedure. This work showed that ROS were involved in MOD, and in particular in myocardial injuries. In our study concerning the impact of CPB on blood antioxidant defences, plasma levels of EPA, an index of these defences, increased. These results suggest that blood antioxidant defences are recruited by this situation. Luyten *et al* (26) also observed this kind of defence stimulation during CPB; gluthation peroxidase and superoxide dismutase activities increased during surgery. In reperfusion, this stimulation of the antioxidant defence system does not seem to be efficient enough to limit the oxidative stress associated with CPB.

In our work, this oxidative stress was associated with increased plasma levels of BDNF. It has been reported that neurotrophins and in particular BDNF could induce the activation of NAD(P)H oxidases and stimulate enzyme activity, and could be the origin of the high levels of oxidative stress that occur during cells degeneration (11, 27). Another study from Wang *et al*. (28) reported on the interaction that exists between BDNF and ROS: increased radical stress induced an increase in BDNF release by endothelial cells of the brain's vascular system.

## CONCLUSION

In conclusion these strong correlations between BDNF and oxidative stress and inflammatory diseases, suggest the existence of links between increased ROS production in patients' plasma and the augmentation of BDNF levels. The role played by this neurotrophin in this complex situation still needs to be elucidated, in particular its cellular origin. It is also necessary to understand whether BDNF has a beneficial or deleterious effect during CPB.
